# Measuring patient‐reported distress from breast magnetic resonance imaging: Development and validation of the MRI‐related distress scale (MRI‐DS)

**DOI:** 10.1002/cam4.70089

**Published:** 2024-08-10

**Authors:** Danbee Kang, Sooyeon Kim, Jiyoon Han, Youngha Kim, Juhee Cho, Jeong Eon Lee, Eun Sook Ko

**Affiliations:** ^1^ Center for Clinical Epidemiology, Samsung Medical Center Seoul South Korea; ^2^ Department of Clinical Research Design and Evaluation, SAIHST Sungkyunkwan University Seoul South Korea; ^3^ Cancer Education Center, Samsung Medical Center Sungkyunkwan University School of Medicine Seoul South Korea; ^4^ Department of Surgery, Samsung Medical Center Sungkyunkwan University School of Medicine Seoul South Korea; ^5^ Department of Radiology, Samsung Medical Center Sungkyunkwan University School of Medicine Seoul South Korea

**Keywords:** breast cancer, distress, magnetic resonance imaging, measurement, patient‐reported outcome, validation

## Abstract

**Objective:**

Although breast magnetic resonance imaging (MRI) is a valuable screening tool, breast MRI testing burden was associated with cancer worry and quality of life. We aimed to develop and validate the MRI‐related distress scale (MRI‐DS) to assess comprehensive distress specifically related to breast MRI.

**Methods:**

We enrolled women aged above 18 years, diagnosed breast cancer, had MRI examination at least one time, and who could speak and read Korean in phase I and enrolled women aged above 18 years, visited outpatient clinic of breast general surgery, had undergone MRI examination at least once, and could speak and read Korean in phase II. We excluded patients who had any physical or psychiatric conditions in both phases. We recruited from a tertiary university‐based hospital in South Korea between April and August 2023.

**Results:**

All 18 items had acceptable levels of item correlation (≥0.30) in the explanatory factor analysis with a four‐factor solution. The fit indices for the four‐factor solution model were good. The discriminant validity of the MRI‐DS had a moderate correlation with general anxiety or quality of life. In the known‐group analysis, those who reported MRI as the most burden breast examination had higher total scores.

**Conclusion:**

The validity of the MRI‐DS has been confirmed as a scale for measuring the specific distress caused by breast MRI. The MRI‐DS is recommended to health professional to communicate with patients with MRI.

**Clinical Implications:**

It can be used to assess the distress associated with MRI screening in breast cancer patients. Physician could use MRI‐DS to discuss the reasons for distress caused by breast MRI screening and to address specific sources of discomfort associated with it.

## 1 BACKGROUND

Breast magnetic resonance imaging (MRI) is a valuable screening tool for individuals at high risk, as it helps in evaluating unknown primary tumors, assessing the extent of local disease, and detecting multicentricity and bilaterality, particularly in those with dense breasts or a history of breast cancer.^1^, ^2^, ^3^, ^4^ Additionally, breast MRI is beneficial for differentiating scars from local recurrences in patients who have undergone breast‐conserving surgery, monitoring the response to neoadjuvant chemotherapy, and evaluating implant integrity.^5^ Recently, annual surveillance with breast MRI has been newly recommended for women with a personal history of breast cancer (PHBC) and dense parenchymal tissue or for breast cancer patients diagnosed by aged 50 years in the updated American College of Radiology 2023.^6^ Korea followed the guideline.^7^

These benefits have led to a significant increase in the use of MRI.^8^ However, the breast MRI testing is significantly associated with cancer worry and reduced quality of life.^9^ Furthermore, only 57.3% of the patients requiring a follow‐up MRI adhered to their follow‐up plans.^10^ Recently, a patient‐reported outcome (PRO) study has been recommended,^11^ but most MRI studies have focused only on diagnostic accuracy, with only few studies having assessed the extent of physical and practical challenges in patient‐centered outcomes.^12^ One of the reasons for this gap is the absence of a suitable PRO measure (PROM) for evaluating patient‐centered issues related to MRI.

While the State–Trait Anxiety Inventory (STAI) and Hospital Anxiety and Depression Scale (HADS) have been used to measure patient anxiety during testing,^13^ these are PROMs designed to measure general anxiety. Consequently, these PROMs are inadequate for assessing the MRI‐related anxiety. The testing morbidity index (TMI) measures the physical and mental aspects of health‐related quality of life (HRQoL) before, during, and after testing.^14^ However, the TMI does not address MRI‐specific issues and cannot accurately access the physical, emotional, or practical issues related to breast MRI.

Therefore, the PROMs have limitations in assessing the distinct impact of breast MRI among breast cancer patients. Thus, we developed and validated a PROM that assessed comprehensive distress specifically related to breast MRI examinations.

## 2 METHOD

We developed an MRI‐related distress scale following the consensus‐based standards for the selection of health measurement instrument (COSMIN) checklist. Phase I encompassed the instrument development phase, which included item development and content validation through cognitive interviews. Phase II involved psychometric validation. To develop this tool, we established an expert group comprising one clinician and five behavioral scientists.

The Institutional Review Board of the Samsung Medical Center, Seoul, Republic of Korea, for the development set (SMC‐2022‐11‐076) approved the study. All participants have been provided informed consent.

### 2.1 Phase I: Instrument development and content validation

#### 2.1.1 Study participants

In Phase I, to evaluate the qualitative validation of the tool, we enrolled women who met the following criteria^1^: aged >18 years,^2^ diagnosed with breast cancer,^3^ had undergone MRI examination at least once, and^4^ could speak and read Korean. Patients with any physical or psychiatric condition that might hinder their ability to complete the interview or questionnaire were excluded. We obtained informed consent from all study participants. We provided the participants a $30 incentive for their participation.

#### 2.1.2 Item development

In this study, we developed the MRI‐related distress scale (MRI‐DS) following the COSMIN checklist. Several steps were assessed in the development of the MRI‐DS. First, an expert group comprising one clinician and five behavioral scientists conducted an extensive literature review. After the literature review, two trained behavioral scientists performed semi‐structured in‐depth interviews with seven breast cancer patients who were aged over 18 years and had experience with MRI at least once. From the qualitative interviews, we identified that patients reported higher distress during breast MRI owing to the breast MRI itself than the general physical or disease‐related issues. The MRI‐DS consists of specific situations within three domains that cause distress for patients when undergoing breast MRI. Physical distress includes specific pain, difficulty in breathing, chest pressure while stepping on or inside the breast MRI machine, and dizziness while getting up from the breast MRI machine. Psychological distress includes anxiety and nervousness while inside the machine, concerns about positioning and breathing that may necessitate retaking, and overall stress related to the breast MRI experience. Practical distress includes the duration of breast MRI, discomfort due to the prone position, noise/vibration from the machine, feeling trapped, and experiencing coldness due to the machine. Based on the literature review and qualitative interviews, 20 items distributed across the three domains (physical distress, *n* = 6; psychological distress, *n* = 6; and practical distress, *n* = 8) were set as the initial version of the MRI‐DS. All questions included 5‐point Likert scale (0 = not at all, 1 = a little, 2 = somewhat, 3 = quite, and 4 = very much) as response option.

#### 2.1.3 Content validation

A cognitive interview was conducted to validate the MRI‐DS content. Based on the grounded theory, we stopped sampling the different patients when the category was theoretical saturated which means that no additional data are being found can develop properties of the category.^15^ In this study, the five patients reported similar instances over and over again, and we considered category was saturated. During the cognitive interview, participants completed the questionnaire. Subsequently, we interviewed them for 30–60 minutes to receive feedback on the content, ease of response, and acceptability of the terminology, phrasing, and response options.

A total of three women with breast MRI examination experience participated in Round 1 of the cognitive interview; one out of three participants was aged over 60 years and had lower literacy levels, with an education level of high school or lower. In Round 1 of the cognitive interview, all patients were confused by the word “examination,” as they believed it encompassed the entire breast examination process, including mammography, ultrasound, and MRI. Thus, we modified it to “breast MRI examination” and included the definition of breast MRI examination. Regarding the questions, “difficulty in breathing or positioning during MRI examination” and “concerns about retaking the MRI examination during the MRI examination,” the patients found it challenging to follow the instructions from the examiner, which resulted in difficulty in breathing and worries about controlling their breathing due to concerns about retaking the MRI examination. Hence, we modified the questionnaire items to a single item include a specific description of the situation. Regarding emotional distress, all patients associated the results of the examination with cancer recurrence or metastasis. Hence, we removed this item.

After modifying the questions, we conducted Round 2 of the cognitive interview with 18 items. Five women with breast MRI examination experience participated in the study, and 40% of the participants were over 60 years of age or had lower literacy levels. All patients fully understood the questions; therefore, the final 18 items within the three domains (physical distress, psychological distress, and practical distress) were used for the psychometric validation.

### 2.2 Phase II: Psychometric validation of MRI‐DS

#### 2.2.1 Study participants

In Phase II, to evaluate the quantitative validation of the tool, we enrolled female patients^1^: aged >18 years,^2^ visited Breast Cancer Center Outpatient Clinic,^3^ had undergone MRI examination at least once, and^4^ could speak and read Korean. We excluded patients with any physical or psychiatric condition that would interfere with the completion of the questionnaire. We enrolled patients from a tertiary university‐based hospital in South Korea between April and August 2023. Informed consent was obtained from all study participants. We provided participants with a $5 incentive for their participation.

#### 2.2.2 Measurement

The discriminant validity of the MRI‐DS was tested using PROMs to assess the Health‐Related Quality of Life (HRQOL), anxiety, and psychological well‐being in comparison with the MRI‐DS. The World Health Organization Quality of Life Assessment Instrument (WHOQOL)‐BREF was used to measure the HRQOL. The WHOQOL‐BREF includes 26 questions within four domains, namely physical (health), psychological, social relationships and environmental, and an overall quality of life and health satisfaction facets.^16^ Based on a scoring manual, the domain scores were calculated by multiplying the mean score of each domain by four. The time span covered the past 2 weeks. To assess anxiety, the State–Trait Anxiety Inventory‐X‐1 (STAI‐X‐1) was used.^17^ We would like to measure fear of recurrence or fear of surveillance with the STAI, to distinguish different types of anxiety from distress caused by the MRI itself which we measured with MRI‐DS and fear of recurrence or fear of surveillance. The STAI‐X‐1 is a 20‐item scale with a 4‐point rating scale (not at all, somewhat, moderately so, and very much so), and a higher total score indicates a higher level of anxiety. The time span covered the previous week. By summing the responses for all items, the total score was obtained with range 20–80. The Korean version of Quality‐of‐Life Cancer Survivors (QOL‐CS‐K) questionnaire was used to measure psychological well‐being. The QOL‐CS‐K questionnaire used to evaluate the quality of life of cancer survivors with including 41 items with four domains (physical, social, psychological, and spiritual well‐being) that are.^18^ Four items, namely fear of future diagnostic tests, fear of a second type of cancer, fear of cancer recurrence, and fear of cancer spreading, were selected from the psychological well‐being domain. The items were rated on a 4‐point Likert scale, with answers ranging from 1 (not at all) to 4 (very much). For these items, higher grades indicate worse psychological well‐being.

Additional sociodemographic and clinical data, including age, sex, marital status, education, yearly household income, employment, residential area, comorbidities, number of breast MRI scans, years since surgery, and tumor stage, were obtained.

#### 2.2.3 Statistical analysis

We performed an exploratory factor analysis (EFA). The internal consistency of each domain was calculated to assess the internal consistency of the MRI‐DS, using Cronbach's *α* and the item‐total correlation of each domain. Good reliability was defined as an *α* value of 0.8 or higher.^19^

Confirmatory factor analysis (CFA) using maximum likelihood without missing values was also performed.^20^ Furthermore, comparative fit index (CFI), standardized root mean square residual (SRMR), and root mean square error of approximation (RMSEA) were calculated. A well‐fitting model typically exhibits CFI >0.9, SRMR <0.08, and RMSEA <0.06.^20^, ^21^, ^22^

We calculated discriminant validity to test the hypothesis of construct validity. We used Pearson's correlations between the MRI‐DS and the WHOQOL‐BREF, STAI‐X‐1, and QOL‐CS‐K. The MRI‐DS, except the psychological distress domain, had weak correlations with WHOQOL‐BREF, STAI‐X‐1, and QOL‐CS‐K (0.30 < *r*), and the psychological distress domain in the MRI‐DS had moderate correlation with QOL‐CS‐K (*r* = 0.30–0.70).

We also performed a known‐group analysis and compared the MRI‐DS based on age group, the number of MRI examinations, and MRI being the most burdensome breast examination.

All analyses were two‐sided, and *p*‐values <0.05 indicated that the value is statistically significant. We used the R software version 3.3.2 (Free Software Foundation, Inc., Boston, MA, USA) for statistical analysis.

## 3 RESULTS

### 3.1 Study participants

A total of 180 patients with experience in breast MRI participated in the survey. The mean age was 51.1 years (standard deviation 9.5) (Table 1). In total, 52.2% patients had several breast MRI examination experiences. All patients completed the questionnaire. The mean completion time for the MRI‐DS survey was approximately 5 min. The participants had received a breast MRI an average of 3 months ago.

**TABLE 1:** Characteristics of the study participants (*N* = 180).

Characteristics*	*N* (%)
	*N* = 180
Age (mean (SD))	51.1 (9.5)
Age group	
20–39	18 (10.0)
40–49	63 (35.0)
50–59	62 (34.4)
≥ 60	37 (20.6)
Marital status	
Married	166 (92.2)
Single	9 (5.0)
Divorced/separated	3 (1.7)
Widowed	1 (0.6)
Living with partner	1 (0.6)
Education	
Less than high school	8 (4.4)
High school graduate	72 (40.0)
More than college/university	100 (55.6)
Employment	
Housewife	119 (66.1)
Employee/self‐employed	46 (25.5)
Sick leave/retired	11 (6.1)
Unemployed	4 (2.2)
Household yearly income	
< $20,000	9 (5.0)
$20,000–$49,999	97 (53.9)
$50,000–$99,999	65 (36.1)
≥ $100,000	9 (5.0)
Residential area: rural area	104 (57.8)
Comorbidities: yes	58 (32.2)
Stage	
0	19 (10.1)
1	62 (34.6)
2	57 (31.8)
3	41 (22.9)
4	1 (0.6)
Year since diagnosis	
<1 year	26 (14.5)
1–2.99 years	105 (58.7)
>3 years	48 (26.8)
Number of MRI examinations: ≥2	94 (52.2)

*Values are presented *n* (%) or mean (SD).

Overall, 97.2% of the patients experienced at least one troublesome symptom on MRI. The most commonly reported issue was bothered due to noise (93.9%), followed by long duration of breast MRI examination (90.6%), and feeling trapped inside (88.3%) (Figure S1).

### 3.2 Validity and reliability

All 18 items satisfied Bartlett's test for sphericity (*p* < 0.01) and the Kaiser–Meyer–Olkin (KMO) test for sampling adequacy (*p* = 0.86). In EFA, all the items had generally acceptable levels of item correlation (≥0.30) (Table 2). The EFA indicated a four‐factor solution with an eigenvalue >1.0 although it was initially designed as a three‐factor solution. The items I feel awkwardness and discomfort when the contrast medium is injected to my body during breast MRI examination, I am concerned the contrast medium might remain in my body after breast MRI examination, and I feel cold inside the machine during breast MRI examination were loaded with same factor solution. These items were initially designed for the practical distress domain. The experts reviewed and concluded that two items, namely “I feel awkwardness and discomfort when the contrast medium is injected to my body during breast MRI examination” and “I am concerned the contrast medium might remain in my body after breast MRI examination” were associated with injection‐related distress during breast MRI examination. Therefore, we separated these two items into “injection‐related distress” domain. Although the item “I feel cold inside the machine during breast MRI examination” also had the same loading as the two injection‐related distress items, the experts thought this item was more closely related to the practical distress domain. Hence, this item was retained in the practical distress domain.

**TABLE 2:** Explanatory factor analysis with four factors (*N* = 180).

Items	FA1	FA2	FA3	FA4	Cronbach alpha
Physical distress					0.82
I have knee pain when stepping on the breast MRI machine			**0.40**		
I have chest pressure during breast MRI examination		0.27	**0.55**		
I have difficulty in breathing, as instructed by the examiner during breast MRI examination	0.26	0.20	**0.65**		
I have shoulder pain due to the prone position during breast MRI examination	0.22		**0.74**		
I have neck pain due to the prone position during breast MRI examination			**0.79**		
I have dizziness when getting up after breast MRI examination	0.20	0.21	**0.63**		
Psychological distress					0.90
I feel nervous being inside the machine during breast MRI examination	0.29	**0.80**			
I feel anxiety being inside the machine during breast MRI examination	0.23	**0.89**			
I am worried that my positioning or breathing during breast MRI examination might be incorrect, leading to a need for retaking	0.26	**0.69**	0.24		
I feel stressed about undergoing breast MRI examination	0.37	**0.63**	0.22	0.23	
Practical distress					0.87
I feel the duration of being inside the machine is too long during breast MRI examination	**0.60**	0.33			
I feel discomfort due to the prone position during breast MRI examination	**0.69**	0.27	0.33		
I feel bothered due to the noise from the machine during breast MRI examination	**0.76**	0.23		0.21	
I feel bothered due to the vibration from the machine during breast MRI examination	**0.79**			0.26	
I feel trapped inside the machine during breast MRI examination	**0.61**	0.43		0.23	
I feel cold inside the machine during breast MRI examination	0.27	0.25	0.23	0.40	
Injection‐related distress					0.82
I feel awkwardness and discomfort when the contrast medium is injected to my body during breast MRI examination	0.23			**0.79**	
I am concerned the contrast medium might remain in my body after the breast MRI examination	0.22			**0.75**	

*Note*: FA means factor loading value in each item. The empty cells in each item row mean the value of factor loading is lower than 0.20 which means weak correlation. The bold value means the highest and signficant factor loading among the factor loading value in each item. MRI is an abbreviation of the magnetic resonance imaging.

**FIGURE 1:**
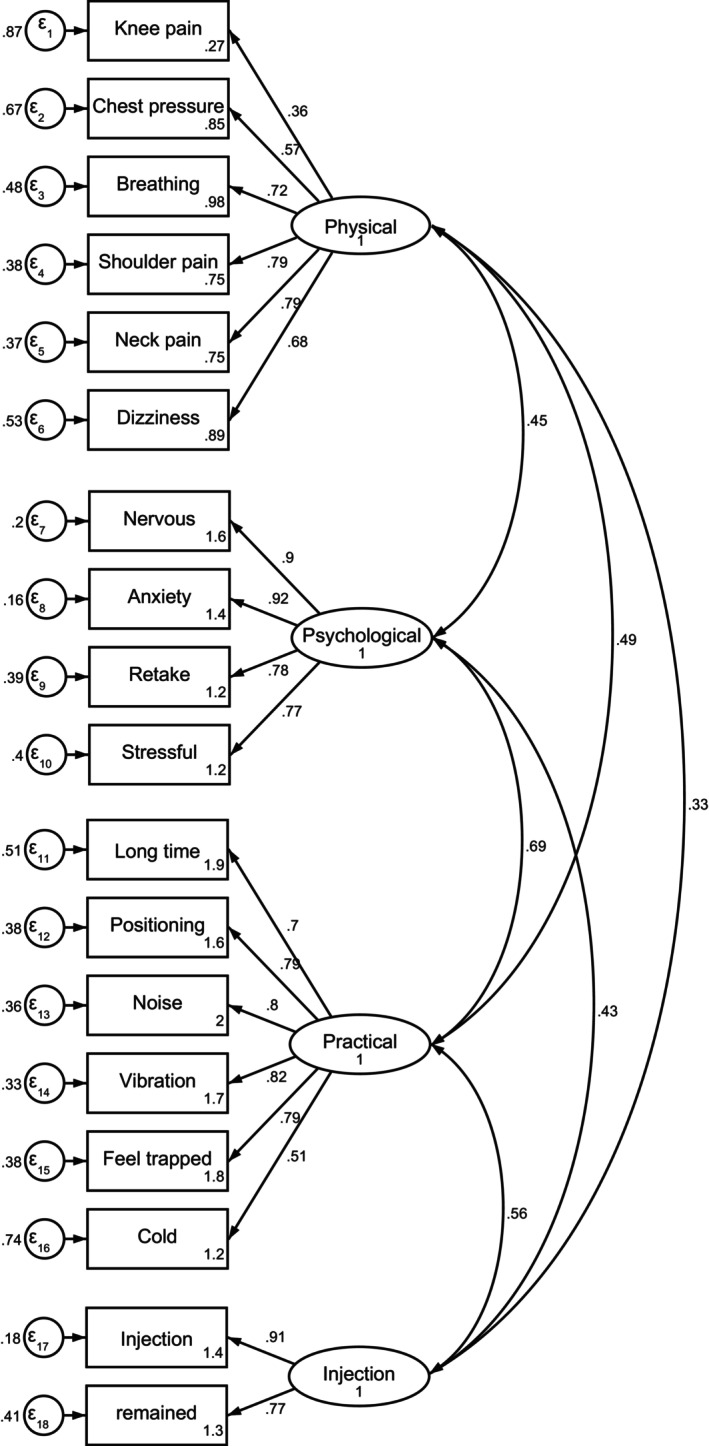
Confirmatory factor analysis of MRI‐related distress (*N* = 180).

The fit indices were good for the four‐factor solution model with a suffering level of CFI of 0.881, an SRMR of 0.070, and an RMSEA of 0.097 (Figure 1). The range of possible scores was from 0 to 72, with the mean (SD) total MRI‐DS being 28.38 (2.8). The floor and ceiling effects were 2.8% and 1.1%, respectively. Cronbach's *α* coefficient ranged from 0.82 to 0.91 for the five scales of the MRI‐DS. Cronbach's *α* coefficient of the total score was 0.91 (Table S1).

The discriminant validity of the MRI‐DS had a moderate correlation with the QOL‐CS‐K questionnaire (range of *r*, 0.34–0.39) (Table 3 and Table S2). In addition, psychological and practical distress domains had moderate correlations with the QOL‐CS‐K questionnaire (range of *r* 0.33–0.39).

**TABLE 3:** Discriminant validity of MRI‐related distress with legacy measures (*N* = 180).

Legacy measures	Total	Physical distress	Psychological distress	Practical distress	Injection‐related distress
STAI‐X‐1	0.27**	0.15*	0.23*	0.30**	0.08
QOL‐CS‐K					
Fear of future diagnostic tests	0.39**	0.17*	0.38**	0.39**	0.24**
Fear of a second type of cancer	0.34**	0.10	0.34**	0.36**	0.23**
Fear of a cancer recurrence	0.35**	0.12	0.36**	0.36**	0.23**
Fear of cancer spreading (metastasis)	0.35**	0.13	0.33**	0.37**	0.22**
WHOQOL‐BREF					
Physical health	−0.17*	−0.09	−0.11	−0.19**	−0.13
Psychological health	−0.10	−0.05	−0.08	−0.11	−0.04
Social relationships	−0.09	0.04	−0.13	−0.14	−0.03
Environment	−0.11	−0.04	−0.10	−0.11	−0.09
Total	−0.14	−0.05	−0.12*	−0.16	−0.09

* *p* < 0.05.

** *p* < 0.01.

In the known‐group analysis, individuals who reported MRI as the most burdensome breast examination had the highest total score (Table 4). Among the domain scores, it was observed that individuals over the age of 60 years tended to have higher physical distress scores, while those who reported MRI as the most burdensome breast examination had higher practical distress scores.

**TABLE 4:** Known‐group analysis of MRI‐related distress with differences in mean of total and domain scores.

Legacy measures	Total	Physical distress	Psychological distress	Practical distress	Injection‐related distress
Age					
<60 years old (*n* = 143)	27.83 (14.16)	**4.57 (4.44)**	7.24 (4.90)	12.48 (6.04)	3.55 (2.41)
≥60 years old (*n* = 37)	30.49 (13.15)	**6.35 (5.01)**	7.65 (4.81)	13.41 (4.91)	3.08 (2.29)
*p‐*value	0.304	**0.035**	0.654	0.389	0.292
Number of MRI examinations					
1 (*n* = 86)	28.80 (13.37)	5.34 (4.75)	7.35 (4.75)	12.57 (5.34)	3.55 (2.29)
>1 (*n* = 94)	27.99 (14.54)	4.56 (4.47)	7.31 (5.00)	12.76 (6.27)	3.36 (2.48)
*p‐*value	0.698	0.262	0.956	0.832	0.605
The most burdensome breast examination					
MRI (*n* = 27)	**33.63 (13.29)**	6.41 (4.89)	8.44 (5.03)	**14.78 (4.62)**	4.00 (2.53)
Mammography (*n* = 152)	**27.38 (13.93)**	4.65 (4.53)	7.13 (4.85)	**12.26 (5.95)**	3.34 (2.35)
*p‐*value	**0.032**	0.068	0.199	**0.038**	0.183

The bold value means it is statistically significant.

## 4 DISCUSSION

This study validated the appropriateness of the MRI‐DS, which included 18 items covering MRI‐related physical, psychological, practical, and injection distress. Overall, 97.2% of the patients experienced at least one troublesome symptom on MRI, especially due to noise and the long duration of the breast MRI examination. The MRI‐DS proved to be more effective in assessing the specific MRI distress than the general HRQOL PROM. Moreover, a correlation existed between the patients' preference for breast MRI and MRI‐DS score.

During our qualitative interviews, the patients reported experiencing physical, psychological, practical, and injection‐related distress associated with MRI screening before, during, and after the procedure. Furthermore, 97.2% of the patients experienced at least one troublesome symptom on MRI. However, current imaging tests only evaluate the accuracy without the patients' perspectives.^23^ Regulatory approval for new tests also prioritizes test accuracy, although the US Food and Drug Administration has a growing interest to consider the additional impacts of tests in such decisions.^23^ Notably, recent studies have reported an the sensitivity of cancer detection was maintained with abbreviated protocol of breast MRI (AB‐MRI) when compared to full dynamic contrast‐enhanced MRI (FP‐MRI).^24^ This could potentially enhance the economic feasibility by decreasing scan and interpretation times. AB‐MRI and FP‐MRI need to be compared in terms of patient‐reported emotional and physical effects,^25^ in addition to assessing their diagnostic performance. This information can be invaluable for shared decision‐making. In such cases, the PRO is important for the patients, caregivers, and the clinicians as the balance of outcomes.^23^ Thus, our MRI‐DS can be used to evaluate these types of tests.

In terms of discriminant validity, the domains in the MRI‐DS showed a weak correlation with the STAI‐X‐1 and WHOQOL‐BREF. This is because the MRI‐DS measures breast MRI‐specific distress and not general psychological and physical issues or the HRQOL. Among the items in the MRI‐DS, the most commonly reported issues were practical distress issues such as bothered due to noise, long duration of breast MRI examination, and feeling trapped inside. The MRI‐DS showed a moderate correlation with four questions related to psychological well‐being in the QOL‐CS‐K questionnaire, specifically regarding apprehensions about future testing, rather than fear of recurrence. Scanxiety is the scan‐associated distress and anxiety experienced before, during, and after cancer‐related imaging/scans, which can be due to the fear of future diagnostic tests and cancer recurrence.^26^ Although previous studies have discussed only on the fear of recurrence, the MRI‐DS additionally focused on the feeling of nervousness and anxiety inside the machine during breast MRI examination and worry about positioning or breathing instead of fear of recurrence, which is an advantage of our MRI‐DS. Furthermore, the MRI‐DS attempted to incorporate all items that represented a unique unmet need, even if they exhibited a low correlation with other items. Although this may have resulted in a relatively low CFI values, this allows MRI‐DS to identify unmet demand for breast MRI and to manage it for the patient's next examination.

In the known‐group analysis, we found that the patients' preference for breast examination was associated with the MRI‐DS score. In our study, participants who reported breast MRI as the most burdensome breast examination had higher total and practical distress scores on the MRI‐DS than those who underwent mammography. This result indicates that the MRI‐DS measures breast MRI‐specific distress and is not related to other types of breast examinations. In addition, as previous studies reported, the specific practical issues related to breast MRI, such as the long duration of examination, noise from the machine, and awkwardness/discomfort of injection, are associated with the patient's preference for breast examination.^27^, ^28^

Several limitations are included in this study. First, we recruited participants from a single hospital using convenience sampling, which may have introduced bias. In particular, we have few patients who were only followed up without breast cancer. However, we tried to enroll patients with characteristics that were representative of the target patient population, including patients with low literacy. In addition, we developed this tool to capture distress due to breast MRI not breast cancer, even if the patients did not have breast cancer, our questionnaire could measure the distress due to breast MRI well. Second, we did not perform test–retest analysis. Our study design did not include any evaluation of clinical stability. However, MRI‐DS items are relatively objective. Third, the convergent validity of the physical, practical, and injection‐related distress domains of the MRI‐DS was not investigated using separate instrument. This was due to reduce the respondents' distress from completing the assessment. The study did not include an existing questionnaire that measures anxiety from MRI screening itself to confirm the convergent validity. However, we confirmed that the MRI‐DS had acceptable validity, which we confirmed through qualitative interviews. Furthermore, MRI‐DS was developed using Korean patients with breast cancer. Nonetheless, previous literature revealed that patients experience for the distress due to breast MRI was similar. Although additional validation study would be necessary, we believe that the MRI‐DS could be a reliable measure for distress due to breast MRI in other countries.

## 5 CONCLUSION

In conclusion, our study confirmed the validity and reliability of the MRI‐DS as a scale for measuring the distress caused by MRI screening for breast cancer. The scale assesses physical, psychosocial, practical, and injection‐related aspects. A future validation study will be necessary to assess the validity of MRI‐DS among participants who do not have breast cancer but require a breast MRI.

## AUTHOR CONTRIBUTIONS

**Danbee Kang:** Conceptualization (equal); formal analysis (equal); methodology (equal); writing – original draft (lead). **Sooyeon Kim:** Conceptualization (equal); formal analysis (equal); methodology (equal); writing – original draft (lead). **Jiyoon Han:** Conceptualization (equal); data curation (equal); methodology (equal). **Youngha Kim:** Conceptualization (equal); methodology (equal). **Juhee Cho:** Conceptualization (equal); methodology (equal). **Jeong Eon Lee:** Conceptualization (equal); methodology (equal); writing – review and editing (equal). **Eun Sook Ko:** Conceptualization (equal); methodology (equal); writing – review and editing (equal).

## FUNDING INFORMATION

The study was funded by the Korean Society of Breast Imaging and Korean Society for Breast Screening (KSBI&KSFBS‐2023‐01) and an Investigator Initiated Research fund from Bayer Korea. This work was also supported by the institution of Quality of Life in Cancer funded by Samsung Fire & Marine Insurance. However, the funding body was not involved in the design of the study, collection, analysis, and interpretation of data, nor the writing the manuscript.

## CONFLICT OF INTEREST STATEMENT

The authors have none to declare.

## ETHICS STATEMENT

This study was approved by the Institutional Review Board of the Samsung Medical Center, Seoul, Republic of Korea, for the development set (IRB no.: SMC‐2022‐11‐076).

## CONSENT

Informed consent was obtained from all participants.

## Supporting information


Data S1:

## Data Availability

The data underlying this article are available in the article.
