# Correction to Measuring patient‐reported distress from breast magnetic resonance imaging: Development and validation of the MRI‐related distress scale (MRI‐DS)

**DOI:** 10.1002/cam4.70219

**Published:** 2024-09-24

Kang, D., Kim, S., Han, J., Kim, Y., Cho, J., Lee, J. E., & Ko, E. S. (2024). Measuring patient‐reported distress from breast magnetic resonance imaging: Development and validation of the MRI‐related distress scale (MRI‐DS). Cancer Medicine, 13(15), e70089. https://doi.org/10.1002/cam4.70089.

On page 1, the following funding information should be added as follows: “Basic Science Research Program through the National Research Foundation of Korea (NRF) funded by the Ministry of Education. Grant Number: 2020R1I1A2074210.”

On page 9, the additional funding information should be added as follows: “Basic Science Research Program through the National Research Foundation of Korea (NRF) funded by the Ministry of Education. Grant Number: 2020R1I1A2074210”.

We apologize for this error.

