# Supplementary material for: Measuring patient‐reported distress from breast magnetic resonance imaging: Development and validation of the MRI‐related distress scale (MRI‐DS)
Source: Cancer Med. 2024 Aug 10;13(15):e70089. doi: 10.1002/cam4.70089 (PMC11316135; doi:10.1002/cam4.70089)
Supplement: Supplementary file 1 — Data S1: [file CAM4-13-e70089-s001.docx]

**Supplementary Table 1.** Description and statistics for MRI distress (N = 180)

**Supplementary Table 2.** Construct validity of psychological and practical distress domain items in the MRI distress questionnaire using the QOL-CS-K questionnaire

**Supplementary Figure 1.** Response distribution of the MRI-related distress questionnaire (N = 180)

**Supplementary Table 1.** Description and statistics for MRI distress (N = 180)

| **Subscales** | **Range** | **Mean (SD)** | **% Floor** | **% Ceiling** | **Cronbach alpha** |
| --- | --- | --- | --- | --- | --- |
| Physical distress | **0-24** | 4.93 | 21.7 | 1.1 | 0.82 |
| Psychological distress | **0-16** | 7.33 | 12.8 | 10.0 | 0.90 |
| Practical distress | **0-24** | 12.67 | 3.9 | 3.3 | 0.87 |
| Injection-related distress | **0-8** | 3.45 | 15.0 | 7.8 | 0.82 |
| **Total** | **0-72** | **28.38** | **2.8** | **1.1** | **0.91** |

**Supplementary Table 2.** Construct validity of psychological and practical distress domain items in the MRI distress questionnaire using the QOL-CS-K questionnaire

| **MRI distress vs QOL-CS-K** | **Fear of future diagnostic tests** | **Fear of a second type of cancer** | **Fear of a cancer recurrence** | **Fear of cancer spreading (metastasis)** |
| --- | --- | --- | --- | --- |
| **MRI-related distress questionnaire** |  |  |  |  |
| **Psychological distress** |  |  |  |  |
| Feeling nervous during examination | **0.31^**^** | 0.27^**^ | 0.25^**^ | 0.24^**^ |
| Feeling anxious during examination | **0.31^**^** | 0.27^**^ | 0.27^**^ | 0.26^**^ |
| Worrying about positioning and breathing | **0.33^**^** | 0.28^**^ | 0.28^**^ | 0.26^**^ |
| Feeling stressed due to breast MRI examination | **0.38**^**^ | **0.38**^**^ | **0.39**^**^ | **0.39**^**^ |
| **Practical distress** |  |  |  |  |
| Long duration of examination | **0.32^**^** | 0.27^**^ | 0.26^**^ | 0.26^**^ |
| Discomforting position | **0.34^**^** | 0.28^**^ | 0.28^**^ | 0.29^**^ |
| Noise from the machine | **0.32^**^** | **0.31^**^** | **0.31^**^** | **0.32^**^** |
| Vibration from the machine | **0.34^**^** | **0.34^**^** | **0.36^**^** | **0.37^**^** |
| Feeling trapped | **0.38^**^** | **0.35^**^** | **0.35^**^** | **0.36^**^** |
| Feeling cold | 0.11 | 0.13 | 0.15^*^ | 0.16^*^ |
| **Injection-related distress** |  |  |  |  |
| Awkwardness/discomfort of injection | 0.21^**^ | 0.18^*^ | 0.19^*^ | 0.18^*^ |
| Concerned of contrast medium remaining | 0.23^**^ | 0.23^**^ | 0.23^**^ | 0.23^**^ |

** P < 0.01; * P < 0.05

**Supplementary Figure 1.** Response distribution of the MRI-related distress questionnaire (N = 180)

**
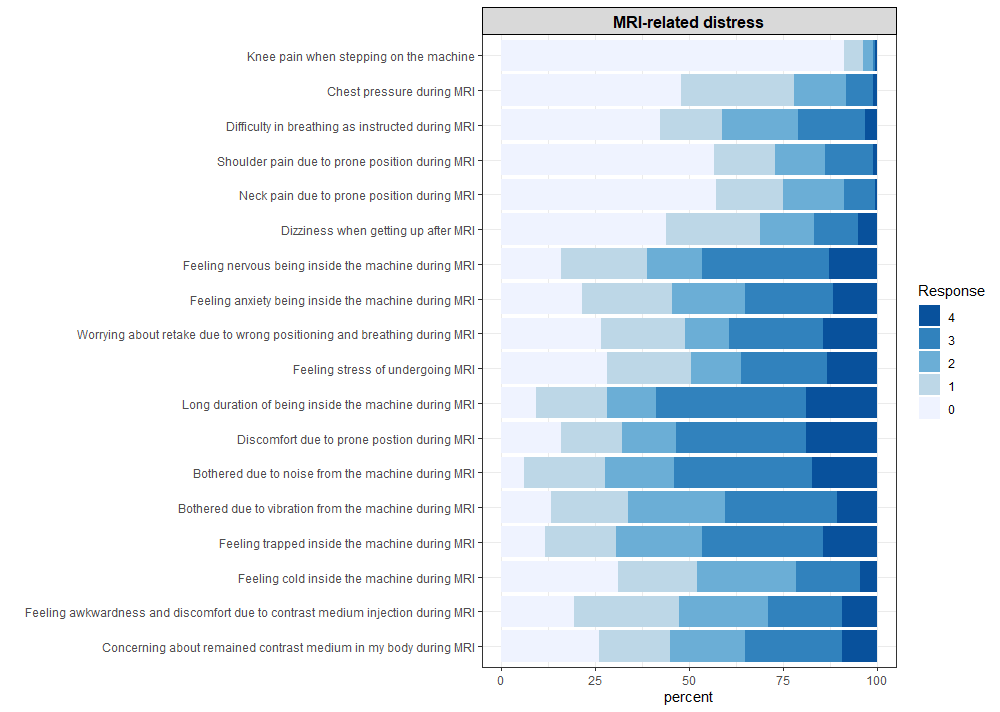
**
